# SOCIAL DETERMINANTS OF COGNITIVE HEALTH: A SCOPING REVIEW

**DOI:** 10.1093/geroni/igad104.0615

**Published:** 2023-12-21

**Authors:** Lingling Zhang, Yao Zhang

**Affiliations:** University of Massachusetts Boston, Boston, Massachusetts, United States; University of Massachusetts Boston, Boston, Massachusetts, United States

## Abstract

Population aging has caused a substantial increase in neurodegenerative disorders, such as Alzheimer’s disease (AD), the fifth leading cause of death for people aged 65 and older. It has posed tremendous challenges to families and society. The existing research shows that postponing AD onset by 5 years would reduce the disease prevalence by half. Therefore, to better understand the modifiable risk factors, including social determinants of health (SDOH) for cognitive health, will be helpful for early prevention and resource allocation. SDOH are classified into five domains (healthcare access and quality, education access and quality, social and community context, economic stability, and neighborhood and built environment). There are studies examining the relationship between one or more domains of SDOH and cognitive health, however, to our knowledge, there is no comprehensive review has been done to synthesize the evidence from all domains of SDOH. This literature review aims to map the existing evidences on social determinants of cognitive health to inform future programs and policies. Twenty-nine studies were included in the review through a systematic search of relevant literature published on peer-reviewed journals from four electronic databases (Pubmed, PsychInfo, CINAHL, and MEDLINE) during 2012-2022. The review summarized a broad range of empirical research on how SDOH affect cognition, as well as revealed the racial disparities in cognitive function, which is worth noting. The favorable SDOH conditions such as better healthcare access, education, social engagement, etc. were proved protective factors for cognitive impairment. Race and racial segregation were found to be the opposite.

